# Sex-specific effects of protein and carbohydrate intake on reproduction but not lifespan in *Drosophila melanogaster*

**DOI:** 10.1111/acel.12333

**Published:** 2015-03-23

**Authors:** Kim Jensen, Colin McClure, Nicholas K Priest, John Hunt

**Affiliations:** 1 Centre for Ecology and Conservation, College of Life and Environmental Sciences, University of Exeter, Cornwall CampusPenryn, TR10 9EZ, UK; 2 Department of Biology and Biochemistry, University of BathBath, BA2 7AY, UK

**Keywords:** caloric restriction, fitness, geometric framework, lifespan, nutrient regulation, reproduction

## Abstract

Modest dietary restriction extends lifespan (LS) in a diverse range of taxa and typically has a larger effect in females than males. Traditionally, this has been attributed to a stronger trade-off between LS and reproduction in females than in males that is mediated by the intake of calories. Recent studies, however, suggest that it is the intake of specific nutrients that extends LS and mediates this trade-off. Here, we used the geometric framework (GF) to examine the sex-specific effects of protein (P) and carbohydrate (C) intake on LS and reproduction in *Drosophila melanogaster*. We found that LS was maximized at a high intake of C and a low intake of P in both sexes, whereas nutrient intake had divergent effects on reproduction. Male offspring production rate and LS were maximized at the same intake of nutrients, whereas female egg production rate was maximized at a high intake of diets with a P:C ratio of 1:2. This resulted in larger differences in nutrient-dependent optima for LS and reproduction in females than in males, as well as an optimal intake of nutrients for lifetime reproduction that differed between the sexes. Under dietary choice, the sexes followed similar feeding trajectories regulated around a P:C ratio of 1:4. Consequently, neither sex reached their nutritional optimum for lifetime reproduction, suggesting intralocus sexual conflict over nutrient optimization. Our study shows clear sex differences in the nutritional requirements of reproduction in *D. melanogaster* and joins the growing list of studies challenging the role of caloric restriction in extending LS.

## Introduction

Modest dietary restriction (DR, a reduction in food intake without malnutrition) has been shown to extend lifespan (LS) across a diverse array of taxa (Mair & Dillin, 2008; Nakagawa *et al*., 2012), and this effect is typically more pronounced in females than in males (Nakagawa *et al*., 2012). Traditionally, the effects of DR on LS have been attributed to caloric restriction (CR, Masoro, 2002, 2005; Partridge & Brand, 2005) and the observed sex differences explained by divergence in the energetic costs of reproduction (Barnes & Partridge, 2003; Bonduriansky *et al*., 2008). In females, the extension of LS with CR is typically explained by the associated reduction in fecundity (Chapman & Partridge, 1998) that enables greater investment in somatic maintenance (Partridge *et al*., 2005). In contrast, the energetic demands of reproduction in males are generally considered to be much lower so that the trade-off between LS and reproduction is less pronounced (Bonduriansky *et al*., 2008). Recent studies, however, have directly challenged these longstanding views by showing that it is the intake of specific nutrients and not calories *per se* that mediates this trade-off and are responsible for extending LS (Lee *et al*., 2008; Maklakov *et al*., 2008; Fanson *et al*., 2009; Fanson & Taylor, 2012; Bruce *et al*., 2013; Solon-Biet *et al*., 2014). Consequently, distinguishing between caloric and specific nutrient effects on LS and reproduction is an essential first step in understanding the mechanisms underlying LS and aging in the sexes, yet one that continues to be the focus of much debate (Simpson & Raubenheimer, 2007, 2009; Piper *et al*., 2011; Tatar, 2011; Fanson & Taylor, 2012; Tatar *et al*., 2014).

The fruit fly model, *Drosophila melanogaster,* has played a central role in the debate over the relative importance of calories vs. specific nutrients to LS (Piper *et al*., 2011; Tatar, 2011; Bruce *et al*., 2013; Tatar *et al*., 2014). Early DR studies on *D. melanogaster* focused almost exclusively on the role of calories in extending LS (Chapman & Partridge, 1998; Masoro, 2002, 2005; Partridge & Brand, 2005), although it is now clear that many of these studies confounded the effects of calories with those of specific nutrients (Piper *et al*., 2011; Tatar, 2011; Tatar *et al*., 2014). More recent work has targeted the specific nutrients that extend LS, and there is good evidence to suggest that protein (P) restriction is largely responsible for the extension in LS observed in many DR studies (Mair *et al*., 2005; Piper *et al*., 2005; Min & Tatar, 2006; Lee *et al*., 2008; Nakagawa *et al*., 2012; Bruce *et al*., 2013). However, despite considerable research, we still know surprisingly little about the exact nutrient (or combination of nutrients) that extends LS in *D. melanogaster* (Tatar *et al*., 2014). A major obstacle in addressing these questions has been the large diversity of different approaches used to restrict dietary intake in this species (Tatar, 2007, 2011; Lee *et al*., 2008; Piper *et al*., 2011; Tatar *et al*., 2014). The most common way to implement DR in *D. melanogaster* has been to restrict the intake of a diet of fixed composition by either limiting access to food or diluting the diet with water or another bulking agent. Studies using this approach have typically used only a small number of diets, have not precisely measured food or nutrient intake, and have used yeast to vary P or overall caloric content (Tatar, 2007; Piper *et al*., 2011). This approach is problematic for a number of reasons. First, using too few diets can make it difficult (if not impossible) to adequately partition the effects of calories and nutrients on LS (Piper *et al*., 2005, 2011; Simpson & Raubenheimer, 2007, 2009; Tatar, 2011). Second, not measuring how much flies actually eat across their lifetime ignores compensatory feeding which can mask any effects of calories or specific nutrients on LS (Fanson *et al*., 2009). Finally, although P is the most abundant macronutrient in yeast, it also contains a variety of micronutrients, essential lipids, sterols, and carbohydrates (Simpson & Raubenheimer, 2009; Tatar, 2011). Thus, while P is the most likely nutrient regulating LS in DR studies using yeast (Mair *et al*., 2005; Min & Tatar, 2006; Lee *et al*., 2008; Skorupa *et al*., 2008), any effects of P are necessarily confounded with these other constituents.

A solution to the above problems can be provided using chemically defined (holidic) diets within the Geometric Framework (GF) for nutrition (Simpson & Raubenheimer, 2012). With the GF approach, the effects of specific intake combinations of multiple nutrients (*n*) can be separated in *n*-dimensional nutritional space by restricting individual animals to an array of diets that differ in nutrient composition and concentration (Simpson & Raubenheimer, 2012). As such, the GF provides a powerful way to partition the effects of specific nutrient and caloric intake on LS and reproduction (Simpson & Raubenheimer, 2007, 2009). Lee *et al*. (2008) recently used this approach to show that the balanced intake of P and C rather than caloric intake *per se* regulates LS and reproduction in female *D. melanogaster*, although hydrolyzed yeast was used as the source of P in this study. Bruce *et al*. (2013) found similar results using liquid and medium diets with both yeast and casein as a source of P, but did not use a full geometric array of diets in their study. No study has yet fully integrated the GF with holidic diets to partition the effects of specific nutrient and caloric intake on LS and reproduction in *D. melanogaster*, even though holidic diets exist and have been applied successfully in this (Lee & Micchelli, 2013; Piper *et al*., 2014) and other species of fruit fly (Fanson & Taylor, 2012). Furthermore, there is a general lack of studies combining the GF with holidic diets to compare the effects of specific nutrient and caloric intake on LS and reproduction between the sexes, with only a single study on field crickets (*Teleogryllus commodus*) taking this approach (Maklakov *et al*., 2008). This work not only confirmed that LS and reproduction are regulated by the balanced intake of P and C and not calories in *T. commodus*, but also demonstrates that the sexes have very different nutritional fitness optima (Maklakov *et al*., 2008). However, male fitness was not measured directly in this study but approximated using calling effort. Thus, more work is needed to establish how the sexes differ in their nutritional optima.

Here, we use the GF approach to distinguish the effects of specific nutrient intake (P and C) from the intake of calories on LS and reproduction in male and female *D. melanogaster*. We conducted two experiments: a no-choice (Experiment 1) and a choice (Experiment 2) experiment. In Experiment 1, we restricted 443 male and 494 female *D. melanogaster* to one of 29 different holidic, liquid diets varying systematically in P and C content, as well as in total nutrient concentration. This experiment provided detailed nutritional landscapes describing the effects of P and C on LS and reproduction in males and females and enabled us to formally compare the nutritional optima for these traits between the sexes. In Experiment 2, 100 flies of each sex were provided with choice between alternate diets differing in P:C ratio and total nutrition. This experiment determines whether flies actively regulate their intake of nutrients when presented with dietary choice and, if so, whether this regulated intake differs between the sexes. These regulated intake points can then be mapped onto the nutritional landscapes from Experiment 1 to ascertain whether the regulation of nutrients shown by the sexes is optimal with regard to LS and reproduction.

## Results

### Experiment 1: No dietary choice on seven nutritional rails at four concentrations

**Fig 1:**
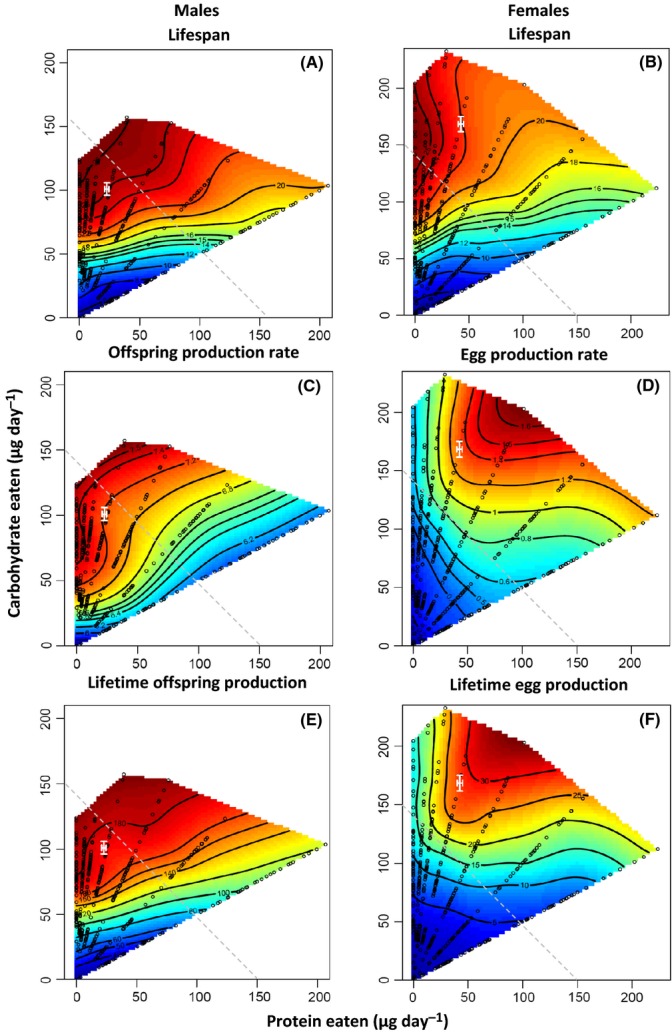
Nonparametric thin-plate spline contour visualizations of the responses surfaces describing the effects of protein and carbohydrate intake on (A) male lifespan (LS), (B) female LS, (C) male offspring production rate, (D) female egg production rate, (E) lifetime offspring production in males, and (F) lifetime egg production in females in *Drosophila melanogaster*. Individual flies were allowed to feed *ad libitum* from one of the 29 liquid foods across their adult LS. Open black circles represent the intake of protein and carbohydrates along each of the seven nutritional rails by individual flies. The regulated intake point (±SE) for flies given the choice between alternate diets (Experiment 2) is mapped on each landscape (in white), after being converted to a daily intake. On each landscape, the gray dashed line represents an ‘isocaloric line’ across the nutritional landscape where a given intake of nutrients yields equal calories. As protein and carbohydrates contain approximately the same amount of energy per unit ingested (∼4 calories per gram), the slope of the isocaloric line is −1.

The nutritional landscapes show that LS and reproduction in *D. melanogaster* are heavily influenced by the intake of P and C (Fig.1, Tables1 & S3). In both sexes, LS was maximized at a high intake of diets containing a low P:C ratio of 1:16 (Fig.1A,B, Fig. S2, Table1). For a given intake of calories, LS decreased with an increasing P:C ratio, which can be visualized by following the isocaloric lines on the nutritional landscapes (Fig.1A,B). Furthermore, LS increased with total caloric intake along each of the nutritional rails, although this was less pronounced as the P:C ratio of diets increased (Fig.1A,B). Consequently, these findings provide little support for the notion that CR extends LS in *D. melanogaster* but do highlight the key role that P and C intake plays in mediating LS. Formal statistical comparison showed little difference between the sexes in the effects of P and C intake on LS (Table S4).

**Table 1:** Linear and nonlinear effects of protein (P) and carbohydrate (C) intake on lifespan and reproduction in male and female *Drosophila melanogaster*

Response variable	Linear effects		Nonlinear effects		
	P	C	P × P	C × C	P × C
Males					
LS					
Gradient ± SE	−0.18 ± 0.03	0.80 ± 0.03	0.03 ± 0.02	−0.17 ± 0.03	0.03 ± 0.05
*t*_442_	5.63	25.39	1.09	5.83	0.53
*P*	0.0001	0.0001	0.28	0.0001	0.60
Offspring production rate					
Gradient ± SE	−0.11 ± 0.05	0.13 ± 0.05	−0.01 ± 0.04	−0.11 ± 0.04	0.06 ± 0.08
*t*_442_	2.34	2.72	0.24	2.41	0.67
*P*	0.02	0.007	0.81	0.02	0.50
Lifetime offspring production					
Gradient ± SE	−0.21 ± 0.04	0.67 ± 0.04	0.01 ± 0.03	−0.18 ± 0.04	0.07 ± 0.06
*t*_442_	5.49	17.74	0.36	5.00	1.13
*P*	0.0001	0.0001	0.72	0.0001	0.26
Females					
LS					
Gradient ± SE	−0.18 ± 0.03	0.74 ± 0.03	0.06 ± 0.02	−0.21 ± 0.02	−0.03 ± 0.04
*t*_493_	5.65	23.32	3.03	9.29	0.74
*P*	0.0001	0.0001	0.003	0.0001	0.46
Egg production rate					
Gradient ± SE	0.26 ± 0.04	0.38 ± 0.04	−0.13 ± 0.03	−0.01 ± 0.03	0.19 ± 0.05
*t*_493_	6.49	9.54	4.44	0.45	3.62
*P*	0.0001	0.0001	0.0001	0.65	0.0001
Lifetime egg production					
Gradient ± SE	0.12 ± 0.04	0.60 ± 0.04	−0.10 ± 0.03	−0.03 ± 0.03	0.23 ± 0.05
*t*_493_	3.44	16.90	3.73	0.97	4.78
*P*	0.001	0.0001	0.0001	0.33	0.0001

The sign of the linear gradients (P or C) describes the direction of the relationship between nutrient intake and the response variable being examined. The nonlinear ‘quadratic’ gradients (P × P and C × C) describe the curvature of this relationship, with a negative gradient indicating a convex relationship (i.e., a peak on the nutritional landscape) and a positive gradient indicating a concave relationship (i.e., a trough on the landscape). The nonlinear ‘correlational’ gradient (P × C) describes how the covariance between nutrients (P and C) influences the response variable. A positive gradient indicates that the response variable increases as the covariance between nutrients increases, whereas a negative gradient indicates that the response variable decreases with an increase in the covariance between nutrients.

Male offspring production rate was maximized at a high intake of diets containing a low P:C ratio (Fig.1C, Table1) and therefore peaked in a similar region on the nutritional landscape as male LS. In contrast, female egg production rate was maximized at a high intake of more P-rich diets, peaking at a P:C ratio of 1:2 (Fig.1D, Table1). As a result, the nutritional landscapes for the daily rate of reproduction differed significantly between the sexes (Table S4). Detailed inspection of the sequential models (Table S4) showed that this sex difference was driven by the linear and nonlinear effects of both nutrients on the daily rate of reproduction. The effects of P and C intake were qualitatively similar when analyzing total lifetime offspring production in both sexes (Fig.1E,F, Table1), and similar sex differences in the nutritional landscapes were found (Table S4). One obvious exception, however, was that the difference in the linear effects of nutrient intake on lifetime reproduction across the sexes was due exclusively to P (Table S4). Importantly, the intake of micronutrients and preservatives contained in our artificial diets did not influence LS or reproduction in the sexes (Table S5), adding further weight to our argument that it is the balanced intake of P and C that influences these traits.

The divergence in the nutritional requirements of the sexes also influences the magnitude of the trade-off between LS and reproduction within the sexes. In males, LS and measures of daily and lifetime reproduction can all be maximized on a high intake of diets with a low P:C ratio, providing little evidence for a trade-off between these traits. In fact, comparison of the nutritional landscapes for male LS and measures of reproduction showed that only the linear component of these surfaces differed significantly due to the stronger positive effects of high C intake on LS than on daily and lifetime reproduction (Table S4). In females, however, the intake of nutrients that maximized LS resulted in suboptimal reproduction and *vice versa*. Consequently, there were significant differences in the linear and nonlinear components of the nutritional landscapes for female LS and measures of reproduction that resulted from the effects of both P and C intake (Table S4). Therefore, in contrast to males, this finding indicates what appears as a trade-off between LS and reproduction in females that is regulated by specific nutrient intake. However, it is important to acknowledge that a trade-off between these traits is not obligatory, particularly as there are regions on the nutritional landscapes that can be traversed where LS remains relatively unchanged but egg production changes considerably (or *vice versa*) (Fig.1).

**Fig 2:**
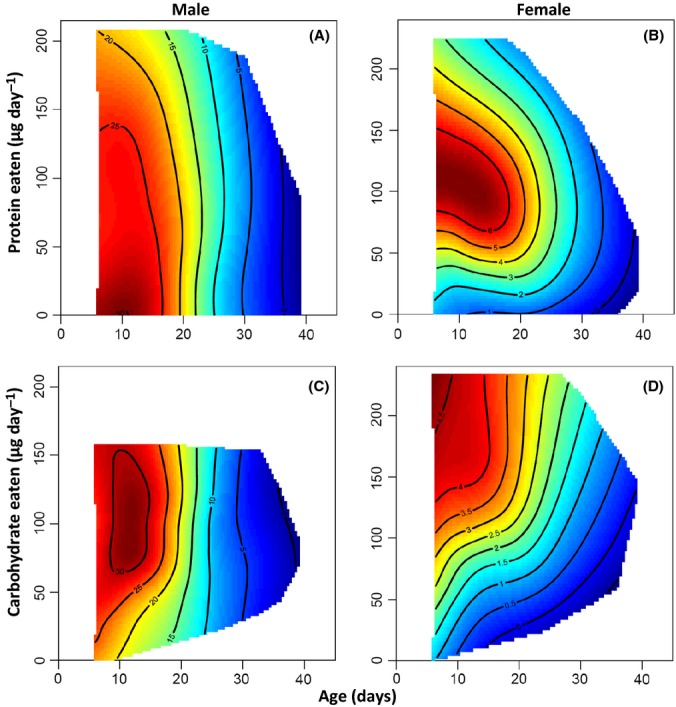
Age-dependent reproduction in relation to protein and carbohydrate intake. Male offspring production (in competition with a 3-day-old Krüppel male with a visible eye mutation) in relation to age and the intake of protein (A) and carbohydrate (C). Female egg production in relation to age and the intake of protein (B) and carbohydrate (D).

**Fig 3:**
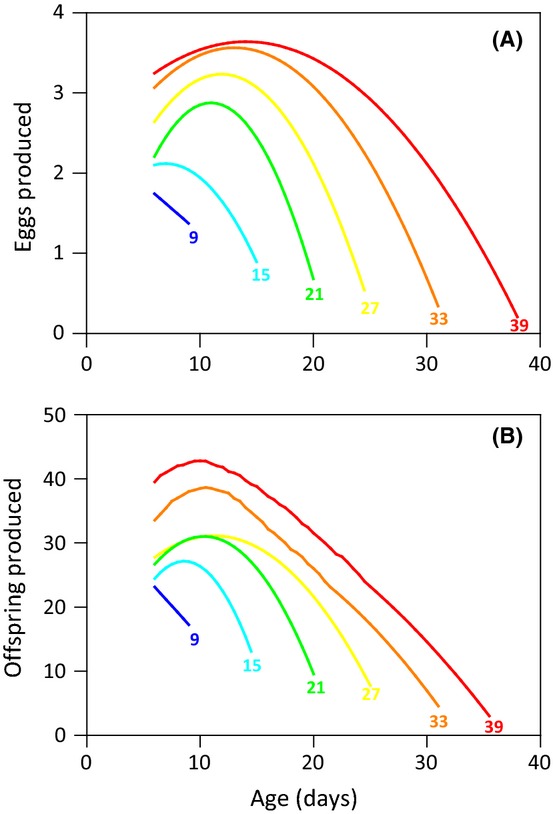
Age-specific trajectories of egg production in females (A) and offspring production in males (B) for cohorts of flies with different lifespans (indicated as numbers next to the trajectory curves).

There was a striking difference between the sexes in age-specific trajectories for reproduction (Fig.2, Table2). Consistent with reproductive aging, reproductive performance decreased with age in both sexes, although this decline was over three times stronger in males than in females (Fig.2, Table2). Reproduction peaked early in life for both sexes, although the range of ages encompassing this peak was much broader in females (∼6–18 days, Figs2B,D and 3A) than in males (∼6–12 days, Figs2A,C and 3B). There was a significant positive interaction between LS and age at maximal reproduction in both sexes (Table2). This reflects longer-lived flies generally having an increased reproductive performance at a given age and that it was only in long-lived individuals where reproduction increased to a peak and then declined (Fig.3). Specific nutrient intake had contrasting effects on reproduction between the sexes with reproduction in females being maximized at a high intake of C and an intermediate intake of P (Fig.2B,D, Table2), whereas male reproduction was maximized at a low intake of P (Fig.2A, Table2). Importantly, there were significant interactions between age and P intake for both sexes (Table2), indicating that the age-specific decline in reproduction is also dependent on the intake of this nutrient. The sign of this interaction, however, differed between the sexes (Table2). In males, this interaction was positive, indicating that the decline in reproduction with age becomes weaker (i.e., less negative) with P intake (Fig.2A), whereas in females, the opposite is true (Fig.2B). Age-specific reproduction did not differ with the intake of C in either sex (Table2).

**Table 2:** Linear and nonlinear response surface gradients for the effects of age, lifespan (LS), protein intake (P), and carbohydrate intake (C) on reproduction in (A) female and (B) male *Drosophila melanogaster*

		Nonlinear effects			
	Linear effects	Age	LS	P	C
A. Female egg production					
Age	−0.184***	−0.094***			
LS	0.069*	0.166***	−0.071*		
P	0.306***	−0.207***	0.177***	−0.152***	
C	0.156***	0.003	0.027	0.064*	−0.032
B. Male offspring production					
Age	−0.560***	−0.308***			
LS	0.274***	0.407***	−0.072*		
P	−0.047*	0.076***	0.005	−0.007	
C	−0.006	−0.004	−0.027	−0.015	−0.004

Gradients were estimated using a GLMM including individual identity as a random effect. Asterisks indicate significance at

*** *P *<* *0.0001,

** *P *<* *0.001, and

* *P *<* *0.05.

### Experiment 2: Measuring nutrient intake under dietary choice

When given dietary choice, both sexes showed a clear preference for the diet containing the highest concentration of C on diet pairs 1 and 3, and females also expressed this preference on diet pair 4 (Fig. S3). Importantly, for both sexes, this resulted in a significantly higher intake of C than expected if flies fed at random from diets for all diet pairs and a significantly lower intake of P for diet pairs 1 and 3 for males and diet pairs 1, 3, and 4 for females (Fig. S4).

**Fig 4:**
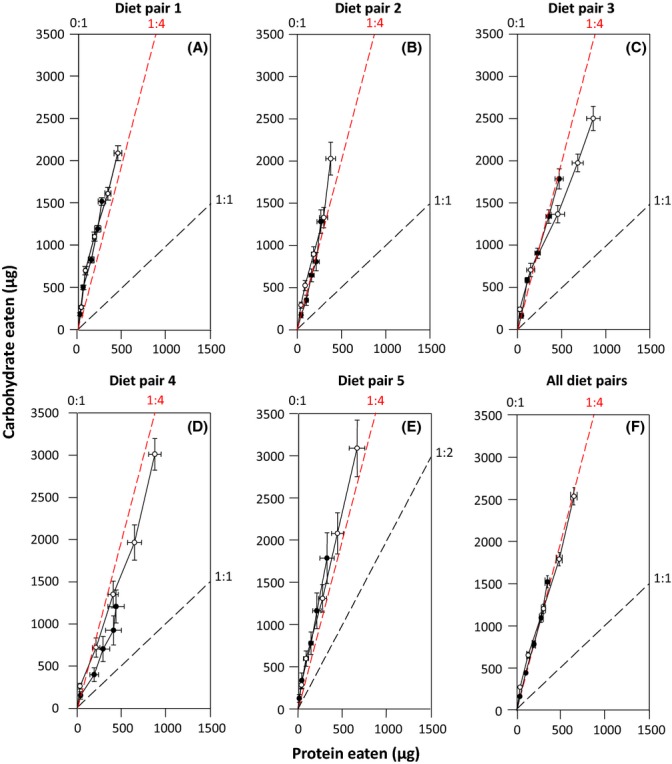
Cumulative intake of protein and carbohydrates (mean ± SE) by male (closed symbols) and female (open symbols) *Drosophila melanogaster* over the first 15 days of adulthood when given the choice between five different diet pairs: (A) diet pair 1: diet 7 vs. diet 27; (B) diet pair 2: diet 7 vs. diet 28; (C) diet pair 3: diet 8 vs. diet 27; (D) diet pair 4: diet 8 vs. diet 28; and (E) diet pair 5: diet 12 vs. diet 28 (see Table S1 for diet compositions). (F) The average cumulative intake of protein and carbohydrates across all diet pairs. The terminal feeding points in (F) represent the regulated intake point for each sex. The P:C ratio of the two diets in each diet pair are provided above the axes, and a black dashed line is provided for the diet that does not have a P:C ratio of 0:1 (which occurs on the y axis). The area between these two P:C ratios represents the region in nutrient space that flies are able to occupy through dietary choice. The red dashed line represents the cumulative intake of nutrients if flies consumed these nutrients in a P:C ratio of 1:4.

There were clear effects of sex, diet pair, and time on the cumulative intake of P and C (Table S5). On average, females had a higher intake of both nutrients than males, flies consumed more nutrients on diet pairs 3, 4, and 5 than on diet pairs 1 and 2, and nutrient intake increased with time (Table S5, Fig.4A–E). There was also a significant interaction between diet pair and time for both nutrients as the cumulative intake trajectories were steeper on some diet pairs (e.g., diet pair 1) than others (e.g., diet pair 4) (Table S5, Fig.4A–E). Importantly, however, the interactions between sex and time and between sex, diet pair, and time were not significant, indicating that the sexes followed the same cumulative feeding trajectories over time (Table S5; Fig.4A–E). Consequently, the regulated intake point for males (P:C = 1:4.43) and females (P:C = 1:3.96) did not differ significantly (sex × P intake: *F*_1,138_ = 0.08, *P *=* *0.79), despite females ingesting significantly more diet than males (sex: *F*_1,138_ = 4.38, *P *=* *0.038; P intake: *F*_1,138_ = 107.19, *P *=* *0.0001). Moreover, neither deviated significantly from a P:C ratio of 1:4 (males: *t*_61_ = 1.90, *P *=* *0.06; females: *t*_79_ = 0.28, *P *=* *0.78). Although in close proximity, the regulated intake point did not completely reside on the nutritional peak for LS or reproduction within any of the sexes, but rather fell midway between the sex-specific reproductive peaks (Fig.1).

## Discussion

It is widely accepted that modest DR extends LS, having been shown in a wide diversity of species, ranging from yeast to primates (Mair & Dillin, 2008), and that this effect is typically stronger in females than in males (Nakagawa *et al*., 2012). Traditionally, the effects of DR on LS have been attributed to CR (Masoro, 2002, 2005; Partridge & Brand, 2005), with the stronger effect in females due to the greater energetic costs of reproduction (Barnes & Partridge, 2003; Bonduriansky *et al*., 2008). Here, we provide empirical evidence that directly contradicts this traditional view. We found that both LS and reproduction in male and female *D. melanogaster*, as well as the apparent trade-off between these two traits within the sexes, are primarily determined by the intake of P and C rather than the intake of calories. Thus, our work adds to the growing list of studies challenging a central role for calories in extending LS (Mair *et al*., 2005; Lee *et al*., 2008; Maklakov *et al*., 2008; Skorupa *et al*., 2008; Fanson *et al*., 2009; Ja *et al*., 2009; Fanson & Taylor, 2012; Nakagawa *et al*., 2012; Bruce *et al*., 2013; Solon-Biet *et al*., 2014) and highlights the utility of applying a robust nutritional framework to disentangle the effects of nutrients and calories on LS (Simpson & Raubenheimer, 2007, 2009).

We found that the intake of P and C had clear effects on LS in *D. melanogaster* and that these effects were largely consistent across the sexes. In both sexes, LS was maximized at a high intake of nutrients at a P:C ratio of approximately 1:16 (Fig.1A,B) and, for a given caloric intake, decreased sharply as the P:C ratio of diets became more P biased. Moreover, LS decreased with a reduced total intake of nutrients (and calories) in both sexes, especially on diets with a low P:C ratio. Our work is therefore consistent with previous studies showing that LS extension under DR in *D. melanogaster* is driven by a restricted intake of P relative to C and not calories *per se* (Mair *et al*., 2005; Lee *et al*., 2008; Skorupa *et al*., 2008; Ja *et al*., 2009; Tatar, 2011; Bruce *et al*., 2013). Our nutritional landscape for female LS (Fig.1B) shows a strong resemblance to an earlier study by Lee *et al*. (2008) on this species using the GF. Although yeast-based diets were used, Lee *et al*. (2008) also found that female LS was maximized at a high intake of nutrients at a P:C ratio of 1:16. Our landscape is also broadly similar to those constructed for female Queensland fruit flies (Q-flies, *Bactrocera tryoni*, Fanson *et al*., 2009; Fanson & Taylor, 2012) and field crickets (*T. commodus*, Maklakov *et al*., 2008), which also show that LS is maximized on diets with a low P:C ratio. However, the exact P:C ratio maximizing female LS is less C biased in *T. commodus* (P:C = 1:8, Maklakov *et al*., 2008) and more C biased in *B. tryoni* than shown for *D. melanogaster* in our study, the magnitude depending on whether yeast-based (P:C = 1:21, Fanson *et al*., 2009) or holidic (P:C = 1:32, Fanson & Taylor, 2012) diets were used. To date, only a single study (Maklakov *et al*., 2008) has compared the effects of P and C intake on LS across the sexes using the GF and found that male and female field crickets have different nutritional optima for LS. Although contrary to our findings for *D. melanogaster*, it is important to note that the sex difference shown for *T. commodus* resulted from LS declining at very high C intake in males but not in females rather than a large shift in the P:C ratio maximizing LS in the sexes (Maklakov *et al*., 2008). That is, male LS was maximized at a P:C ratio of 1:5, but the peak on the nutritional landscape was broad and clearly overlapped the P:C ratio maximizing female LS (1:8) in this species (Maklakov *et al*., 2008). Collectively, these studies provide compelling support for the lethal protein hypothesis (Simpson & Raubenheimer, 2007, 2009) and suggest that the extension of LS on low P:C ratio diets may be a widespread pattern across the animal kingdom. Indeed, a recent meta-analysis across DR studies showed that the effect of P intake (in relation to the intake of other macronutrients) on LS is much larger than that of caloric intake (Nakagawa *et al*., 2012).

In contrast to LS, we found strong divergence in the effects of P and C on reproduction between the sexes in *D. melanogaster* (Fig.1C,D). Male offspring production rate was maximized at the same P:C ratio as LS (1:8, Fig.1C), whereas female egg production was maximized at a high intake of diets with a P:C ratio of 1:2 (Fig.1D). This difference in the nutritional requirements for reproduction most likely reflects the divergence in the reproductive strategies of the sexes. In most species, the intensity of sexual selection acting on males is far greater than on females because fathers contribute less to each offspring than mothers do (Bonduriansky *et al*., 2008). As a result, males often face intense competition for access to mates and individuals with the most elaborate sexual traits or behaviors are frequently the most successful (Bonduriansky *et al*., 2008). To fuel these costly traits and behaviors, males require a high intake of C as this provides an abundant source of energy that can be accessed rapidly after digestion (Maklakov *et al*., 2008; South *et al*., 2011). For example, producing an advertisement call is metabolically demanding (Kavanaugh, 1987) and a key determinant of mating success in male *T. commodus* (Hunt *et al*., 2004), and this sexual trait is maximized at a P:C ratio of 1:8 (Maklakov *et al*., 2008). In contrast, females typically do not have to compete for matings and their reproductive success is largely determined by the number of eggs they produce. In many insect species, egg production is closely linked to nutrition through a neurohormonal feedback system and P intake plays an important role in stimulating oogenesis and regulating vitellogenesis (Wheeler, 1996). It is therefore not surprising that females typically require a higher intake of P than males to maximize their reproductive success (Maklakov *et al*., 2008). Indeed, our finding that the rate of egg production in *D. melanogaster* is maximized at a P:C ratio of 1:2 is highly consistent with previous GF studies on this species (P:C = 1:2, Lee *et al*., 2008; Reddiex *et al*., 2013), as well as on female field crickets (P:C = 1:1, Maklakov *et al*., 2008) and Q-flies (P:C = 1:2.3, Fanson *et al*., 2009; 1:1, Fanson & Taylor, 2012).

The sex difference we show in the effects of P and C intake on reproduction in *D. melanogaster* is also highly consistent with the divergence observed in male and female field crickets (Maklakov *et al*., 2008). However, our findings contradict a recent study on *D. melanogaster* which showed that both male and female reproductions were maximized at a P:C ratio of 1:2 (Reddiex *et al*., 2013). Although male reproduction was measured as the number of offspring sired under competitive conditions in both studies, this was measured over the entire LS of each male in our study but only over the first 4 days of adulthood in Reddiex *et al*. (2013). In *D. melanogaster*, an intermediate intake of P over the first 12 days of adulthood provides males with an advantage in sperm competition, most likely through an increase in the production of sperm and/or Acps (Fricke *et al*., 2008). It is therefore possible that the difference between our study and Reddiex *et al*. (2013) reflects the greater need for P early in adulthood to promote sexual maturation. Indeed, data from our choice experiment shows that male P intake increases over the first 9 days of adulthood before leveling off, whereas C intake continued to increase over the 15-day feeding period, and the intake of P is mirrored by male reproductive performance (Fig. S5). In contrast, the same alignment of P intake and reproductive performance was not observed in females (Fig. S5).

Evolutionary theories of aging predict that senescence in reproductive performance evolves because the strength of natural selection declines with age (Williams, 1966; Bonduriansky *et al*., 2008). Although there is evidence of reproductive aging in a diversity of species measured in both the laboratory and field (Bonduriansky *et al*., 2008), there are surprisingly few studies examining how nutrition influences this process (Maklakov *et al*., 2009). Our analysis of age-specific reproduction in *D. melanogaster* shows clear reproductive aging in both sexes, although this was more than three times stronger in males than in females. Furthermore, we found that the intake of P significantly influenced reproductive aging within both sexes, although differently between the sexes (Table2). In females, reproductive aging increased in strength with the intake of P, whereas in males, it decreased in strength with the intake of this nutrient (Fig.2, Table2). Together, these findings contradict the pattern of reproductive aging shown in male and female field crickets (Maklakov *et al*., 2009). In *T. commodus*, females show strong reproductive aging while male reproductive performance increases with age (Maklakov *et al*., 2009). Furthermore, even though reproductive performance was nutrient dependent in both sexes, reproductive aging was not (Maklakov *et al*., 2009). Consistent with this study, however, we found that longer-lived individuals had increased reproductive performance at a given age and that it was in these individuals where the increase in reproduction followed by a decline was most pronounced (Fig.2 and 3). This pattern was more pronounced in male than in female flies (Table2), while the opposite was true in *T. commodus* (Maklakov *et al*., 2009). One likely explanation for the different pattern of reproductive aging observed in male flies and crickets is the way that male reproductive performance is measured in these two species. Reproductive performance in male *T. commodus* was measured as the time spent calling to attract a mate and therefore does not examine the competitive ability of males in obtaining a mating or their ability to sire offspring once mating has occurred. This may overestimate reproductive performance and make it difficult to detect reproductive aging in males.

The trade-off between reproduction and LS due to the competing demands for resources is central to evolutionary theories of aging (Williams, 1966; Kirkwood & Holliday, 1979; Barnes & Partridge, 2003; Partridge *et al*., 2005). Traditionally, calories have been viewed as the limiting resource that regulates this trade-off (Gadgil & Bossert, 1970) and the greater energetic demands of reproduction have been used to explain why this trade-off is typically stronger in females than in males (Barnes & Partridge, 2003; Bonduriansky *et al*., 2008). In agreement with this view, we show that there are opposing effects of nutrients on LS and reproduction in female but not in male *D. melanogaster*. However, we show that this apparent trade-off is mediated by the intake of P and C rather than calories. The nutritional landscapes for LS and reproduction in males were similar, with both traits being maximized on a high intake of diets with a low P:C ratio (Fig.1A,C,E). In contrast, female LS and reproduction were maximized at very different regions in nutrient space: LS increased with C intake across isocaloric rails on the nutritional landscape (Fig.1B), whereas reproduction was maximized at an intermediate P:C ratio and declined as diets became more P or C biased (Fig.1D,F). Thus, the intake of nutrients that maximizes LS in females is suboptimal for reproduction and *vice versa*. This finding is consistent with the classic Y-model of life-history trade-offs (Van Noordwijk & de Jong, 1986) which states that both LS and reproduction cannot be maximized because increasing reproductive effort diverts essential resources away from somatic maintenance and LS. It is important to note, however, that LS and egg production can both be maximized on a low P:C ratio diet in female *D. melanogaster* when the diet is supplemented with methionine, suggesting that a trade-off between LS and reproduction is not inevitable but could be caused by imbalances between individual amino acids (Grandison *et al*., 2009). However, it is important to note that the lack of detectable costs in this study may reflect the fact that costs could lie in other unmeasured aspects of fly reproductive performance (i.e., offspring quality). Furthermore, at least one follow-up study has reported contrasting effects of methionine, showing that this amino acid reduced LS but had little effect on reproduction (Zajitschek *et al*., 2013).

Alternatively, the observed differences in the nutritional landscapes for LS and reproduction in females may arise because there are direct costs to P ingestion (Simpson & Raubenheimer, 2009; Fanson *et al*., 2012). The intake of P is clearly required for egg production in *D. melanogaster*, but above a certain intake, P has a detrimental effect on LS and reproduction. Importantly, this pattern was also shown in males, suggesting this effect is not contingent on the allocation of resources to reproduction *per se* but rather a direct consequence of over-ingesting this nutrient. The most likely candidates responsible for this effect are the elevated production of toxic nitrogenous wastes (Singer, 2003) or mitochondrial reactive oxygen species that are known to increase with P consumption (Sanz *et al*., 2004; Ayala *et al*., 2007). A recent study on mice suggests that the reduction in LS and the elevation in markers of poor cardio-metabolic health observed on diets with a high P:C ratio result from a high expression of mTOR (Solon-Biet *et al*., 2014). In general, there is growing support for the lethal P hypothesis (Simpson & Raubenheimer, 2009; Fanson *et al*., 2012) with similar declines in LS and reproduction at high P intake being reported in all species where the GF approach has been used (Lee *et al*., 2008; Maklakov *et al*., 2008; Fanson *et al*., 2009; Fanson & Taylor, 2012). Moreover, Fanson *et al*. (2012) recently provided direct empirical support for this hypothesis by showing that Q-flies varying in reproductive status (mated, virgin, and sterilized females and virgin males) all experienced a similar decrease in LS with increasing P intake, even though sterilized females and males require little P for reproduction. Further tests of this nature are needed to separate the relative importance of the Y-model and lethal P hypothesis to the trade-off between LS and reproduction in *D. melanogaster*.

Optimal foraging theory predicts that animals will evolve foraging mechanisms that maximize their fitness (Stephens & Krebs, 1986). While early studies focused exclusively on the intake of energy (Stephens & Krebs, 1986), there is now considerable evidence demonstrating that animals can also regulate their intake of specific nutrients to maximize fitness (e.g., Simpson *et al*., 2004; Jensen *et al*., 2012). We found that our best estimate of fitness (lifetime reproduction) was highly divergent across the sexes in *D. melanogaster*, suggesting that fitness will be best maximized by the sexes regulating their intake of P and C independently. Despite this, we failed to see divergence in the feeding trajectories of the sexes under dietary choice with males and females both regulating their intake of nutrients at a P:C ratio of 1:4 (Fig.4F). While comparable with earlier studies on female *D. melanogaster* (P:C = 1:2, Lee *et al*., 2008) and Q-flies (1:3, Fanson *et al*., 2009), this pattern of nutrient regulation was not optimal for either sex in our study (Fig.1E,F). Furthermore, the lack of divergence between the sexes is consistent with the pattern shown in field crickets where there is clear sex-specific nutritional optima for lifetime reproduction, but the sexes shared a common feeding trajectory at a P:C ratio of 1:2.96 (Maklakov *et al*., 2008). Maklakov *et al*. (2008) argued that this shared dietary choice prevents the sexes from reaching their sex-specific nutrient optima: a process referred to as intralocus sexual conflict (ISC) (Bonduriansky & Chenoweth, 2009). ISC arises whenever there are sex-specific optima for a trait that is expressed in both sexes, but the shared genetic basis for this trait prevents the sexes from evolving independently to their optima (Bonduriansky & Chenoweth, 2009). Definitive evidence of ISC over nutrient optimization therefore requires showing that the sexes (i) have different nutritional optima and (ii) share a common genetic basis for their dietary preferences, which can be characterized by a strong and positive intersexual genetic correlation (*r*_MF_) (Bonduriansky & Chenoweth, 2009). To date, only a single study has measured these key parameters and concluded there was little evidence for ISC over nutrient optimization in *D. melanogaster* (Reddiex *et al*., 2013). This result, however, is not altogether unsurprising given that nutrient intake was only measured over a very short time period (4 days) which is likely to explain the minor differences in the nutritional landscapes across the sexes (relative to the differences we show) and a *r*_MF_ for P intake that did not differ statistically from zero (Reddiex *et al*., 2013). Like the study of Maklakov *et al*. (2008), our results are highly suggestive that ISC over nutrient optimization exists in *D. melanogaster*, but further work is needed to estimate *r*_MF_ over an appropriate timeframe before this process can be viewed as an important evolutionary constraint in this species.

In conclusion, our study clearly shows that it is the balanced intake of P and C rather than calories *per se* that extends LS and maximizes reproduction in *D. melanogaster*. It is important to note, however, that although the provision of liquid diets in microcapillary tubes (referred to as the CAFÉ feeding assay, Ja *et al*., 2007) is an accurate way to measure the intake of nutrients in *Drosophila* (Deshpande *et al*., 2014), measurements of LS and reproduction using this assay are typically reduced compared to more commonly applied laboratory assays using solid diets. That is, while average LS and reproduction observed in our study were comparable to other studies on *D. melanogaster* using the CAFÉ assay (e.g., Carvalho *et al*., 2005; Lee *et al*., 2008; Lushchak *et al*., 2014), these values were much lower than reported on agar-based holidic medium diets (e.g., Lee & Micchelli, 2013; Piper *et al*., 2014) or more traditional agar-based yeast medium diets (e.g., Mair *et al*., 2005; Bruce *et al*., 2013). Despite this, however, it is important to note that the nutritional landscape for LS that we report is similar in shape to those published for *D. melanogaster* using solid media (Lee *et al*., 2008; Piper *et al*., 2011), suggesting that the general P:C effect on LS appears robust. Until we understand why flies live shorter when housed individually on liquid diets, results of the CAFÉ assay should be interpreted with a degree of caution and would likely benefit by being used in conjunction with alternate approaches, such as radioisotope labeling (Deshpande *et al*., 2014).

## Experimental procedures

### Fly stock and maintenance

Flies originated from the Dahomey stock (provided by Stuart Wigby, University of Oxford) and were maintained with overlapping generations in two large population cages (1 m^3^) at 25 °C under a 12:12 L:D photoperiod at a population size of approximately two thousand flies per cage. Flies were cultured on standard sugar–yeast medium (80 g oat semolina, 138 g treacle, 16 g yeast, 7.5 g agar, 2 g methyl paraben, 206 mL propionic acid, and 21 mL phosphoric acid in 1.6 L of water). Flies were reared at a density of 45–55 larvae per vial (25 × 95 mm) with flies from eight vials per week contributing to each population cage. Our stock cultures of flies were maintained according to this protocol for 2 years prior to use in our experiment.

All experimental flies were reared following this same protocol and housed in individual vials within 2 h of eclosion to adulthood. Once individually housed, flies were randomly allocated to experiments and to individual diets (Experiment 1) or diet pairs (Experiment 2) within experiments.

### Artificial liquid diets and measuring nutrient intake

A total of 29 artificial liquid diets (Fig. S1, Table S1) were constructed that varied in protein (P) and carbohydrate (C) ratio, as well as in total nutrition (P + C), using a modified version of the protocol outlined in Fanson & Taylor (2012). This produced diets along seven discrete nutritional rails (0:1, 1:16, 1:8, 1:4, 1:2, 1:1, 2:1) (Fig. S1). Protein consisted of 18 different free amino acids (Table S2) mixed according to the protocol outlined in Chang *et al*. (2001), whereas carbohydrates consisted of sucrose. All diets also contained a fixed amount of cholesterol (4.00 g L^−1^), RNA from yeast (10.00 g L^−1^), Vanderzant vitamin mixture (3.60 g L^−1^), Wesson salt mixture (10.00 g L^−1^), and methyl paraben (1.50 g L^−1^). Our artificial diets therefore differed from those used by Fanson & Taylor (2012) where micronutrients were provided in direct proportion to the amount of protein contained in the diets. Our artificial diets were designed to cover the same nutrient space as the yeast- and sucrose-based diets used by Lee *et al*. (2008) and the holidic diets used by Fanson & Taylor (2012). We also included a single diet lacking protein and carbohydrates (Diet 29, Table S1) to root our nutritional landscapes at the origin. While the majority of our diets readily dissolved into a homogeneous solution, this was not possible for a few of the higher concentration diets.

Liquid diets were provided in either a single (Experiment 1) or a pair (Experiment 2) of 5-μL microcapillary tubes (Drummond Microcaps, Alpha Laboratories, Hampshire, UK) (Ja *et al*., 2007; Lee *et al*., 2008), and the consumption of diets was measured over 3-day feeding intervals. For both sexes, microcapillary tubes were removed late in the afternoon of the third day while male and female reproduction was assessed (see below) and replaced with freshly filled tubes the following morning. For each feeding period, the amount of diet in microcapillary tubes was measured to the nearest 0.25 mm before and after each feeding period using a precision ruler (Lee *et al*., 2008). To control for the evaporation of diets, two microcapillary tubes per diet were established in individual vials (without flies) during each feeding period and diet loss measured as outlined above. Control vials were maintained together with experimental vials during experiments. Total diet consumption was estimated in each feeding period by subtracting the volume of diet in tubes before and after feeding and then subtracting the mean loss of diet due to evaporation estimated from the two control tubes from this value. Mean (±SE) absolute evaporation on each of the diets is provided in Table S1. Across our 29 diets, average evaporation (as a percentage of total diet consumed) was 11.28 (SE: 1.41, range: 2.94–30.86)%. Total diet consumption was converted to an intake of specific nutrients by multiplying this volume by the specific nutrient concentration of the diet (Table S1). We placed a small square (2 × 2 cm) of moistened black absorbant paper in the bottom of all vials to serve as an additional water source and also as an oviposition site for females (Lee *et al*., 2008). This paper was replaced every 3 days at feeding and at no stage did this paper dry out during our experiments. This feeding regime ensured that males and females had the same access to food and water throughout the experiment.

### Experiment 1: No dietary choice on seven nutritional rails at four concentrations

To characterize and compare the linear and nonlinear effect of P and C intake on LS and reproduction in male and female *D. melanogaster*, 18 flies of each sex were assigned at random on their day of eclosion to each of the 29 artificial diets. Individual flies were fed and reproduction assessed every 3 days over the duration of their lifetime. Starting at day 3, all flies were paired with a virgin, 3-day-old mating partner for 12 h taken at random from the stock culture. This was repeated across the lifetime of all experimental flies, with a new 3-day-old virgin mating partner being used each time. Female reproduction was assessed by counting the number of eggs oviposited on the moistened absorbent paper. To provide a more biologically relevant measure of male reproductive success, we measured the number of offspring produced by each experimental male when in competition against a virgin, 3-day-old male harboring a dominant eye-shaped mutation (Krüppel) that allowed us to easily assign the paternity of offspring. The Krüppel mutation had been backcrossed into our Dahomey stock for 28 generations prior to use in our experiment. After 12 h, the Krüppel male and female were removed and the female established in an individual tube with 7 mL of ‘jazz mix’ diet (Fisher Scientific, Loughborough, UK) for 14 days, after which tubes were frozen and offspring-phenotyped and counted. The survival of all experimental flies was monitored daily to measure LS. As LS differed across diets, reproduction was provided across both the lifetime of flies (male lifetime offspring production and female lifetime egg production) and a daily measure (male offspring production rate and female egg production rate). The later was calculated by dividing lifetime reproduction by LS. In total, we had data available on nutrient intake, LS, and reproduction for 443 male and 494 female flies. Flies that died before their first mating or escaped during the course of the experiment (28 females and 79 males) were excluded from our analyses.

#### Statistical analysis

We used a multivariate response-surface approach (Lande & Arnold, 1983) to estimate the linear and nonlinear (i.e., quadratic and correlational) effects of P and C intake on our response variables (LS, lifetime and daily reproduction) for each sex. As recommended by Lande & Arnold (1983), the linear gradients for P and C intake were estimated from a model containing only the linear terms, whereas the nonlinear gradients for these nutrients were estimated from a model that contained both the linear and nonlinear terms. Nutritional landscapes were visualized using nonparametric thin-plate splines implemented in the fields package of R (version 2.13.0, GNU General Public License).

We used a sequential model building approach to assess whether the linear and nonlinear effects of protein and carbohydrate intake differed for our response variables within and across the sexes (South *et al*., 2011). When significant linear (i.e., P and C) or quadratic (i.e., P × P and C × C) effects were detected in these sequential models, univariate tests were used to determine which of the nutrients were responsible (South *et al*., 2011). As our response variables were measured in different units, they were standardized using a *Z*-transformation prior to analysis (South *et al*., 2011).

We used a general linear mixed models (GLMM) to fit multivariate response surfaces for the effects of P and C intake on age-specific reproduction in the sexes (Maklakov *et al*., 2009). This model included individual identity as a random effect, P and C intake, LS and age as fixed effects, and reproduction as the response variable and therefore accounts for both within-individual and between-individual variation in age-specific reproduction in the same model (van de Pol & Verhulst, 2006). As described above, we estimated the linear and nonlinear gradients in each sex using separate GLMMs and the response surfaces were visualized using nonparametric thin-plate splines. Our multivariate responses surfaces, sequential models, and GLMMs were all analyzed using ibm spss software (version 20), IBM, Portsmouth, UK.

### Experiment 2: Measuring nutrient intake under dietary choice

To examine how male and female *D. melanogaster* regulate their intake of nutrients when provided dietary choice, 20 flies of each sex were assigned at random on their day of eclosion to each of five different diet pairs that varied in both the ratio of protein to carbohydrates and total nutrition (P:C(total nutrition)): pair 1: 1:1(180 g L^−1^) vs. 0:1(180 g L^−1^), pair 2: 1:1(180 g L^−1^) vs. 0:1(360 g L^−1^), pair 3: 1:1(360 g L^−1^) vs. 0:1(180 g L^−1^), pair 4: 1:1(360 g L^−1^) vs.0:1 (360 g L^−1^), and pair 5: 1:2(360 g L^−1^) vs. 0:1(360 g L^−1^). This corresponds to diets 7, 8, 12, 27, and 28 in Table S1 and provides good coverage of nutrient space on the nutritional landscape (Fig. S1). As outlined above, the consumption of both diets in each pair was measured every 3 days for 15 days posteclosion and the same mating scheme was followed as in Experiment 1. In total, we had data available on nutrient intake under dietary choice for 66 males and 86 females. Flies that died before 15 days or escaped during the course of the experiment (four females and 24 males) were excluded from our analyses.

#### Statistical analysis

To determine whether flies consumed significantly more of one diet in each pair, we compared the total absolute consumption of both diets using paired *t*-tests. To determine the implications of dietary choice for nutrient intake, we first calculated the expected intake of P and C for each fly, assuming they consumed diets at random. This expected intake was then subtracted from the observed intake of these nutrients and the difference compared to a mean of zero (i.e., expected if flies were eating at random) using a one-sample *t*-test (South *et al*., 2011).

To determine whether the intake of nutrients changed with diet pair and sex over the duration of our feeding experiment, we analyzed the cumulative intake of P and C across the five feeding periods using repeated-measures ANOVA. We included sex, diet pair, and time as main effects in this model, plus all possible interaction terms. Significant interactions between sex and time and/or between sex, diet pair, and time would indicate that the sexes follow different feeding trajectories over time. As this overall model showed significant differences in nutrient intake across diet pairs (Table S3), we conducted post hoc analysis within each of the pairs using a reduced model that included only sex, time, and their interaction. This same model was used to compare the average cumulative intake of nutrients across diet pairs.

The regulated intake point for each sex, defined as the point in nutritional space to which animals regulate when provided with dietary choice (Simpson *et al*., 2004), was calculated as the mean total intake of P and C across all diet pairs. To determine whether the regulated intake point differed between the sexes, we used ANCOVA including sex (main effect), total P intake (covariate), and their interaction as model terms and total C intake as the response variable. A significant sex by protein intake interaction would indicate that the sexes have different regulated intake points. We also compared the regulated intake point for each sex to a P:C ratio of 1:4, by calculating and then subtracting the expected intake of nutrients by each fly if consuming nutrients at this ratio from the observed intake of nutrients and comparing this difference to a mean of zero using a one-sample *t*-test.

## Supporting Information

**Fig. S1** Geometric presentation of the P and C concentration of the holidic diets used in our experiments. **Fig. S2** Survival curves for male and female flies across diets differing in P:C ratio and total nutrient concentration. **Fig. S3** Mean (±SE) intake of diets in each of the diet pairs for male and female flies. **Fig. S4** Mean (±SE) difference in the intake of P and C from the intake of these nutrients if male and female flies fed at random from the two diets in each diet pair. **Fig. S5** Mean (±SE) intake of P and C, as well as reproductive performance, of male and female flies when given dietary choice. **Table S1** Nutrient contents and mean evaporation of the holidic diets used in our experiments. **Table S2** Amino acid mixture used to vary protein content in our holidic diets. **Table S3** Mean estimates of nutrient intake, LS, and reproduction for the sexes on each diet in Experiment 1. **Table S4** Sequential models comparing the effects of P and C intake of LS and reproduction in the sexes. **Table S5** The effect of P, C, and micronutrient (M) intake on LS and reproduction in the sexes. **Table S6** The effect of sex, diet pair, and time on the intake of P and C under dietary choice.
