# Supplementary material for: Sex-specific effects of protein and carbohydrate intake on reproduction but not lifespan in Drosophila melanogaster
Source: Aging Cell. 2015 Mar 23;14(4):605–15. doi: 10.1111/acel.12333 (PMC4531074; doi:10.1111/acel.12333)
Supplement: Supplementary file 1 [file acel0014-0605-sd1.docx]

**Table S1.** Nutrient contents and ratios of the 29 artificial liquid diets used in our experiments. We also provide the mean (± SE) evaporation on each of the diets, measured over a 3 day feeding cycle for two replicate microcapillary tubes per diet.

| **Diet** | **Protein**  **(P)**  **(g/(L water))** | **Carbohydrate**  **(C)**  **(g(/L water))** | **Concentration**  **(P + C)**  **(g/(L water))** | **Ratio**  **(P:C)**  **(g:g)** | **% P**  **(g/(100g liquid diet))** | **% C**  **(g/(100g liquid diet))** | **Mean evaporation (μL ± SE)** |
| --- | --- | --- | --- | --- | --- | --- | --- |
| 1 | 30.00 | 15.00 | 45.00 | 2:1 | 2.87 | 1.44 | 1.72 ± 0.18 |
| 2 | 60.00 | 30.00 | 90.00 | 2:1 | 5.51 | 2.75 | 1.30 ± 0.14 |
| 3 | 120.00 | 60.00 | 180.00 | 2:1 | 10.17 | 5.08 | 1.04 ± 0.20 |
| 4 | 240.00 | 120.00 | 360.00 | 2:1 | 17.65 | 8.82 | 0.70 ± 0.12 |
| 5 | 22.50 | 22.50 | 45.00 | 1:1 | 2.15 | 2.15 | 1.30 ±0.12 |
| 6 | 45.00 | 45.00 | 90.00 | 1:1 | 4.13 | 4.13 | 1.53 ± 0.15 |
| 7 | 90.00 | 90.00 | 180.00 | 1:1 | 7.63 | 7.63 | 0.59 ± 0.08 |
| 8 | 180.00 | 180.00 | 360.00 | 1:1 | 13.24 | 13.24 | 0.55 ± 0.05 |
| 9 | 15.00 | 30.00 | 45.00 | 1:2 | 1.44 | 2.87 | 1.45 ± 0.18 |
| 10 | 30.00 | 60.00 | 90.00 | 1:2 | 2.75 | 5.50 | 1.31 ± 0.19 |
| 11 | 60.00 | 120.00 | 180.00 | 1:2 | 5.08 | 10.17 | 0.99 ± 0.16 |
| 12 | 120.00 | 240.00 | 360.00 | 1:2 | 8.82 | 17.65 | 0.71 ± 0.07 |
| 13 | 9.00 | 36.00 | 45.00 | 1:4 | 0.86 | 3.44 | 1.31 ± 0.12 |
| 14 | 18.00 | 72.00 | 90.00 | 1:4 | 1.65 | 6.61 | 1.09 ± 0.11 |
| 15 | 36.00 | 144.00 | 180.00 | 1:4 | 3.05 | 12.20 | 0.94 ± 0.10 |
| 16 | 72.00 | 288.00 | 360.00 | 1:4 | 5.29 | 21.18 | 0.73 ± 0.10 |
| 17 | 5.00 | 40.00 | 45.00 | 1:8 | 0.48 | 3.83 | 1.40 ± 0.15 |
| 18 | 10.00 | 80.00 | 90.00 | 1:8 | 0.92 | 7.34 | 1.00 ± 0.10 |
| 19 | 20.00 | 160.00 | 180.00 | 1:8 | 1.69 | 13.56 | 0.70 ± 0.10 |
| 20 | 40.00 | 320.00 | 360.00 | 1:8 | 2.94 | 23.53 | 0.71 ± 0.11 |
| 21 | 2.65 | 42.35 | 45.00 | 1:16 | 0.25 | 4.05 | 1.49 ± 0.11 |
| 22 | 5.29 | 84.71 | 90.00 | 1:16 | 0.49 | 7.77 | 1.04 ± 0.10 |
| 23 | 10.59 | 169.41 | 180.00 | 1:16 | 0.90 | 14.36 | 0.67 ± 0.09 |
| 24 | 21.18 | 338.82 | 360.00 | 1:16 | 1.56 | 24.91 | 1.21 ± 0.14 |
| 25 | 0.00 | 45.00 | 45.00 | 0:1 | 0.00 | 4.31 | 1.30 ± 0.13 |
| 26 | 0.00 | 90.00 | 90.00 | 0:1 | 0.00 | 8.26 | 1.10 ± 0.09 |
| 27 | 0.00 | 180.00 | 180.00 | 0:1 | 0.00 | 15.25 | 0.66 ± 0.08 |
| 28 | 0.00 | 360.00 | 360.00 | 0:1 | 0.00 | 26.47 | 0.93 ± 0.08 |
| 29 | 0.00 | 0.00 | 0.00 | 0:0 | 0.00 | 0.00 | 1.69 ± 0.18 |

All liquid foods also contained cholesterol (4.00 g/L), RNA from yeast (10.00 g/L), Vanderzant vitamin mixture (3.60 g/L), Wesson salt mixture (10.00 g/L), and methyl paraben (1.50 g/L).

**Table S2.** Amino acid mixture used to vary protein content in our artificial liquid diets. We also provide the amino acid mixture used in the holidic, medium diet of Piper *et al.* (2004) for comparison. Although there are subtle differences in the concentration of most amino acids across diets, the major difference is that the liquid diet used in our experiments does not contain L-asparagine or L-glutamine.

| **Amino acid** | **g/(100g)** | **g/(100g) in Piper *et al.* (2014)** |
| --- | --- | --- |
| L-alanine | 4.87 | 9.32 |
| L-arginine | 6.67 | 2.13 |
| L-asparagine | 0 | 4.53 |
| L-aspartic acid | 7.05 | 4.53 |
| L-cysteine | 2.55 | 0.27 |
| L-glutamic acid | 24.51 | 26.63 |
| L-glutamine | 0 | 6.66 |
| Glycine | 5.62 | 8.52 |
| L-histidine | 2.85 | 2.66 |
| L-isoleucine | 3.52 | 0.48 |
| L-leucine | 6.75 | 0.32 |
| L-lysine | 3.67 | 5.06 |
| L-methionine | 1.72 | 2.13 |
| L-phenylalanine | 4.42 | 3.46 |
| L-proline | 7.80 | 4.00 |
| L-serine | 4.87 | 5.06 |
| L-threonine | 3.37 | 5.33 |
| L-tryptophan | 1.80 | 1.33 |
| L-tyrosine | 3.00 | 0.11 |
| L-valine | 4.95 | 7.46 |

**Table S3.** The mean (±SE) intake of protein (P) and carbohydrate (C), lifespan (LS) and reproductive performance (RP) of male and female flies on our 29 artificial, holidic diets. The composition of these diets is presented in Table S1.

| **Diet** | **♀ mean P intake**  **(μg/day)** | **♀ mean C intake**  **(μg/day)** | **♂ mean P intake**  **(μg/day)** | **♂ mean C intake**  **(μg/day)** | **Mean ♀ LS**  **(days)** | **Mean ♂ LS**  **(days)** | **Mean ♀ RP**  **(eggs/day)** | **Mean ♂ RP (offspring/day)** |
| --- | --- | --- | --- | --- | --- | --- | --- | --- |
| 1 | 26.48 ± 1.51 | 13.24 ± 0.76 | 27.77 ± 1.52 | 13.88 ± 0.76 | 6.06 ± 0.25 | 6.19 ± 0.21 | 0.26 ± 0.07 | 7.38 ± 1.05 |
| 2 | 49.96 ± 4.80 | 24.98 ± 2.40 | 57.26 ± 2.77 | 28.63 ± 1.39 | 6.67 ± 0.44 | 8.20 ± 0.33 | 0.44 ± 0.10 | 5.64 ± 0.84 |
| 3 | 119.87 ± 4.20 | 59.94 ± 2.10 | 119.04 ± 8.74 | 59.52 ± 4.38 | 8.59 ± 0.55 | 15.63 ± 1.69 | 0.72 ± 0.15 | 5.31 ± 0.70 |
| 4 | 150.40 ± 11.29 | 75.20 ± 5.65 | 169.90 ± 6.94 | 84.95 ± 3.47 | 13.53 ± 1.09 | 16.93 ± 1.49 | 0.64 ± 0.16 | 6.35 ± 0.29 |
| 5 | 21.22 ± 1.45 | 21.22 ± 1.45 | 20.70 ± 0.88 | 20.70 ± 0.88 | 6.81 ± 0.31 | 6.76 ± 0.25 | 0.48 ± 0.14 | 5.67 ± 1.05 |
| 6 | 40.68 ± 1.75 | 40.68 ± 1.75 | 42.69 ± 0.73 | 42.69 ± 0.73 | 8.22 ± 0.34 | 10.77 ± 0.71 | 0.35 ± 0.09 | 7.37 ± 0.84 |
| 7 | 92.31 ± 6.27 | 92.31 ± 6.27 | 93.95 ± 3.21 | 93.95 ± 3.21 | 16.14 ± 2.15 | 20.94 ± 1.77 | 0.79 ± 0.18 | 6.95 ± 0.71 |
| 8 | 113.94 ± 8.05 | 113.94 ± 8.05 | 92.74 ± 4.63 | 92.74 ± 4.63 | 17.00 ± 1.77 | 21.69 ± 1.73 | 0.92 ± 0.17 | 6.44 ± 0.44 |
| 9 | 15.16 ± 0.58 | 30.32 ± 1.17 | 14.03 ± 0.46 | 28.06 ± 0.92 | 7.17 ± 0.31 | 7.64 ± 0.40 | 0.32 ± 0.08 | 6.74 ± 0.86 |
| 10 | 32.43 ± 1.01 | 64.86 ± 2.03 | 30.93 ± 1.17 | 61.86 ± 2.33 | 10.11 ± 0.82 | 17.93 ± 1.47 | 0.47 ± 0.10 | 7.56 ± 0.39 |
| 11 | 46.62 ± 2.69 | 93.23 ± 5.38 | 43.09 ± 1.33 | 86.18 ± 2.66 | 18.50 ± 1.84 | 23.88 ± 1.26 | 0.93 ± 0.12 | 6.94 ± 0.71 |
| 12 | 71.01 ± 3.74 | 142.02 ± 7.49 | 54.29 ± 3.49 | 108.59 ± 6.98 | 18.35 ± 2.12 | 23.20 ± 1.47 | 1.20 ± 0.23 | 7.39 ± 0.67 |
| 13 | 8.61 ± 0.60 | 34.44 ± 2.39 | 8.11 ± 0.24 | 32.43 ± 0.98 | 7.56 ± 0.55 | 8.00 ± 0.43 | 0.24 ± 0.07 | 8.36 ± 1.11 |
| 14 | 20.39 ± 0.31 | 81.55 ± 1.25 | 18.43 ± 0.50 | 73.74 ± 1.99 | 19.56 ± 1.15 | 24.19 ± 0.98 | 0.41 ± 0.06 | 7.13 ± 0.57 |
| 15 | 26.92 ± 1.95 | 107.66 ± 7.81 | 21.90 ± 0.97 | 87.59 ± 3.86 | 21.06 ± 1.71 | 24.15 ± 1.16 | 0.67 ± 0.12 | 6.89 ± 0.42 |
| 16 | 33.14 ± 2.30 | 132.56 ± 9.19 | 24.14 ± 2.03 | 96.54 ± 8.11 | 17.31 ± 1.93 | 24.06 ± 1.40 | 1.24 ± 0.23 | 5.82 ± 0.69 |
| 17 | 4.66 ± 0.25 | 37.25 ± 2.02 | 4.27 ± 0.15 | 34.15 ± 1.14 | 9.18 ± 0.63 | 8.94 ± 0.47 | 0.35 ± 0.12 | 6.15 ± 0.79 |
| 18 | 9.82 ± 0.76 | 78.52 ± 6.07 | 10.74 ± 0.16 | 85.92 ± 1.30 | 17.89 ± 1.92 | 24.36 ± 1.24 | 0.45 ± 0.10 | 8.13 ± 0.68 |
| 19 | 14.37 ± 0.98 | 114.96 ± 7.84 | 11.89 ± 0.52 | 95.10 ± 4.13 | 21.00 ± 1.89 | 24.46 ± 1.86 | 0.73 ± 0.12 | 5.62 ± 0.74 |
| 20 | 20.49 ± 1.54 | 163.91 ± 12.33 | 10.24 ± 0.80 | 81.95 ± 6.41 | 25.13 ± 2.13 | 23.77 ± 1.72 | 0.83 ± 0.12 | 7.64 ± 0.47 |
| 21 | 2.28 ± 0.15 | 36.54 ± 2.47 | 2.44 ± 0.07 | 39.07 ± 1.12 | 9.06 ± 0.67 | 9.88 ± 0.33 | 0.11 ± 0.02 | 7.47 ± 0.57 |
| 22 | 5.36 ± 0.14 | 85.73 ± 2.31 | 4.92 ± 0.10 | 78.71 ± 1.64 | 21.76 ± 1.37 | 26.13 ± 1.17 | 0.39 ± 0.06 | 8.23 ± 0.67 |
| 23 | 6.90 ± 0.33 | 110.45 ± 5.30 | 6.07 ± 0.17 | 97.08 ± 2.77 | 20.50 ± 1.71 | 26.12 ± 1.32 | 0.61 ± 0.11 | 7.39 ± 0.45 |
| 24 | 7.63 ± 0.78 | 122.03 ± 12.49 | 3.44 ± 0.40 | 55.11 ± 6.48 | 24.64 ± 1.51 | 25.93 ± 1.29 | 0.69 ± 0.12 | 6.90 ± 0.57 |
| 25 | 0.00 ± 0.00 | 49.09 ± 2.77 | 0.00 ± 0.00 | 46.69 ± 0.70 | 13.11 ± 1.63 | 11.13 ± 0.52 | 0.14 ± 0.06 | 7.97 ± 0.67 |
| 26 | 0.00 ± 0.00 | 86.81 ± 2.40 | 0.00 ± 0.00 | 78.82 ± 1.58 | 24.00 ± 1.59 | 24.63 ± 1.57 | 0.20 ± 0.03 | 7.02 ± 0.50 |
| 27 | 0.00 ± 0.00 | 103.38 ± 6.04 | 0.00 ± 0.00 | 89.93 ± 4.15 | 23.56 ± 1.40 | 23.31 ± 1.79 | 0.36 ± 0.07 | 8.48 ± 1.55 |
| 28 | 0.00 ± 0.00 | 149.34 ± 10.31 | 0.00 ± 0.00 | 78.62 ± 7.54 | 22.47 ± 1.69 | 22.87 ± 1.22 | 0.47 ± 0.12 | 7.03 ± 0.62 |
| 29 | 0.00 ± 0.00 | 0.00 ± 0.00 | 0.00 ± 0.00 | 0.00 ± 0.00 | 5.17 ± 0.13 | 5.18 ± 0.13 | 0.02 ± 0.01 | 4.19 ± 0.66 |

**Table S4.** Sequential *F*-tests comparing the effects of P and C intake on lifespan and reproduction within and between the sexes in *D. melanogaster*. The sequential *F*-tests test the differences in the sign and strength of the linear and nonlinear regression gradients across different response variables or the sexes. When significant differences in linear or quadratic regression gradients were detected, univariate tests were used to determine whether this overall effect was due to the intake of P or C (or both).

|  | ***SS*_R_** | ***SS*_C_** | ***DF*_1_** | ***DF*_2_** | ***F*** | ***P*** |
| --- | --- | --- | --- | --- | --- | --- |
| **Males vs. Females** | |  |  |  |  |  |
| *Lifespan vs. Lifespan* | | | | | | |
| Linear | 413.36 | 412.46 | 2 | 931 | 1.02 | 0.36 |
| Quadratic | 356.37 | 354.77 | 2 | 927 | 2.09 | 0.12 |
| Correlational | 354.74 | 354.45 | 1 | 925 | 0.75 | 0.39 |
| *Offspring production rate vs. Egg production rate* | | | | | | |
| Linear | 859.71 | 803.93 | 2 | 931 | 32.30 | 0.0001^A^ |
| Quadratic | 797.51 | 788.22 | 2 | 927 | 5.47 | 0.004^B^ |
| Correlational | 780.08 | 774.02 | 1 | 925 | 7.24 | 0.007 |
| *Lifetime offspring production vs. Lifetime egg production* | | | | | | |
| Linear | 577.01 | 553.04 | 2 | 931 | 20.18 | 0.0001^C^ |
| Quadratic | 546.24 | 533.54 | 2 | 927 | 11.04 | 0.0001^D^ |
| Correlational | 522.08 | 519.82 | 1 | 925 | 4.02 | 0.045 |
| **Males** |  |  |  |  |  |  |
| *Lifespan vs. Offspring production rate* | | | | | | |
| Linear | 704.79 | 611.05 | 2 | 880 | 67.50 | 0.0001^E^ |
| Quadratic | 590.64 | 589.00 | 2 | 876 | 1.22 | 0.30 |
| Correlational | 588.52 | 588.46 | 1 | 874 | 0.08 | 0.77 |
| *Lifespan vs. Lifetime offspring production* | | | | | | |
| Linear | 440.88 | 436.53 | 2 | 880 | 4.39 | 0.013^F^ |
| Quadratic | 404.77 | 404.74 | 2 | 876 | 0.03 | 0.97 |
| Correlational | 404.07 | 403.94 | 1 | 874 | 0.29 | 0.59 |
| **Females** |  |  |  |  |  |  |
| *Lifespan vs. Egg production rate* | | | | | |  |
| Linear | 670.07 | 605.33 | 2 | 982 | 52.51 | 0.0001^G^ |
| Quadratic | 588.02 | 553.98 | 2 | 978 | 30.04 | 0.0001^H^ |
| Correlational | 550.57 | 544.31 | 1 | 976 | 11.22 | 0.001 |
| *Lifespan vs. Lifetime egg production* | | | | | |  |
| Linear | 552.24 | 528.97 | 2 | 982 | 21.60 | 0.0001^I^ |
| Quadratic | 510.82 | 483.56 | 2 | 978 | 27.57 | 0.0001^J^ |
| Correlational | 478.62 | 470.33 | 1 | 976 | 17.18 | 0.0001 |

Univariate tests: ^A^ = P: *F*_1,931_ = 35.52, *P* = 0.0001, C: *F*_1,931_ = 15.61, *P* = 0.0001; ^B^ = PxP: *F*_1,927_ = 5.69, *P* = 0.017, CxC: *F*_1,927_ = 4.50, *P* = 0.034; ^C^ = P: *F*_1,931_ = 40.35, *P* = 0.0001, C: *F*_1,931_ = 1.72, *P* = 0.19; ^D^ = PxP: *F*_1,927_ = 6.26, *P* = 0.013, CxC: *F*_1,927_ = 14.40, *P* = 0.0001; ^E^ = P: *F*_1,880_ = 1.18, *P* = 0.28, C: *F*_1,880_ = 131.32, *P* = 0.0001; ^F^ = P: *F*_1,880_ = 0.37, *P* = 0.54, C: *F*_1,880_ = 7.00, *P* = 0.008; ^G^ = P: *F*_1,982_ =73.88, *P* = 0.0001, C: *F*_1,982_ = 49.01, *P* = 0.0001; ^H^ = PxP: *F*_1,978_ = 22.08, *P* = 0.0001, CxC: *F*_1,978_ = 35.61, *P* = 0.0001; ^I^ = P: *F*_1,982_ = 40.05, *P* = 0.0001, C: *F*_1,982_ = 8.17, *P* = 0.004; ^J^ = PxP: *F*_1,978_ = 15.31, *P* = 0.0001, CxC: *F*_1,978_ = 37.77, *P* = 0.0001;

**Table S5.** Linear and nonlinear effects of protein (P) and carbohydrate (C) intake on lifespan and reproduction in male and female *Drosophila melanogaster*, when controlling for the intake of micronutrients (M) from our liquid diets. Micronutrients contained in the diet consist of cholesterol, RNA from yeast and Vanderzant vitamin and Wesson salt mixtures, as well as the preservative methyl paraben, in the concentrations provides in Table S2. In our analysis, we sum these dietary constituents and refer to them collectively as “micronutrients”. Following the protocol outlined in Sentinella *et al.* (2013), we include the daily intake of micronutrients as a linear term in our response surface model to examine the effects of P and C on lifespan and reproduction after removing the effects of micronutrient intake on these response variables. The intake of M did not have a significant effect on lifespan or reproduction in either sex. Furthermore, removal of the effects of M intake on these response variables had little effect on the linear and nonlinear gradients for P and C intake (by comparison to Table 1).

|  | **Linear effects** | | |  | **Nonlinear effects** | | |
| --- | --- | --- | --- | --- | --- | --- | --- |
| Response variable | P | C | M |  | P × P | C × C | P × C |
| **Males** |  |  |  |  |  |  |  |
| *Lifespan* |  |  |  |  |  |  |  |
| Gradient ± SE | -0.15 ± 0.03 | 0.75 ± 0.03 | -0.05 ± 0.04 |  | 0.02 ± 0.02 | -0.16 ± 0.03 | 0.03 ± 0.05 |
| *t*_442_ | 4.56 | 22.47 | 1.17 |  | 0.78 | 5.82 | 0.56 |
| *P* | 0.0001 | 0.0001 | 0.88 |  | 0.44 | 0.0001 | 0.58 |
| *Offspring production rate* |  |  |  |  |  |  |  |
| Gradient ± SE | -0.12 ± 0.05 | 0.14 ± 0.05 | 0.04 ± 0.05 |  | 0.04 ± 0.04 | -0.11 ± 0.05 | 0.03 ± 0.08 |
| *t*_442_ | 2.41 | 2.66 | 0.78 |  | 0.90 | 2.34 | 0.33 |
| *P* | 0.02 | 0.008 | 0.43 |  | 0.37 | 0.02 | 0.74 |
| *Lifetime offspring production* |  |  |  |  |  |  |  |
| Gradient ± SE | -0.14 ± 0.04 | 0.63 ± 0.04 | -0.07 ± 0.04 |  | 0.04 ± 0.03 | -0.17 ± 0.04 | 0.05 ± 0.06 |
| *t*_442_ | 3.52 | 15.48 | 1.85 |  | 1.27 | 4.84 | 0.80 |
| *P* | 0.0001 | 0.0001 | 0.07 |  | 0.21 | 0.0001 | 0.42 |
| **Females** |  |  |  |  |  |  |  |
| *Lifespan* |  |  |  |  |  |  |  |
| Gradient ± SE | -0.18 ± 0.03 | 0.75 ± 0.03 | 0.04 ± 0.03 |  | 0.07 ± 0.02 | -0.21 ± 0.02 | -0.03 ± 0.04 |
| *t*_493_ | 5.73 | 22.78 | 1.08 |  | 3.05 | 9.18 | 0.78 |
| *P* | 0.0001 | 0.0001 | 0.28 |  | 0.002 | 0.0001 | 0.44 |
| *Egg production rate* |  |  |  |  |  |  |  |
| Gradient ± SE | 0.26 ± 0.04 | 0.37 ± 0.04 | -0.06 ± 0.04 |  | -0.14 ± 0.03 | -0.02 ± 0.03 | 0.20 ± 0.05 |
| *t*_493_ | 6.59 | 8.85 | 1.37 |  | 4.55 | 0.64 | 3.76 |
| *P* | 0.0001 | 0.0001 | 0.17 |  | 0.0001 | 0.53 | 0.0001 |
| *Lifetime egg production* |  |  |  |  |  |  |  |
| Gradient ± SE | 0.12 ± 0.04 | 0.60 ± 0.04 | -0.02 ± 0.04 |  | -0.10 ± 0.03 | -0.03 ± 0.03 | 0.23 ± 0.05 |
| *t*_493_ | 3.47 | 16.15 | 0.50 |  | 3.78 | 1.07 | 4.84 |
| *P* | 0.001 | 0.0001 | 0.62 |  | 0.0001 | 0.29 | 0.0001 |

**Table S6.** Repeated Measures ANOVA examining the effect of sex (male or female), diet pair (pair 1 to 5) and time (day 0 to 15) on the cumulative intake of protein and carbohydrates when provided with dietary choice. As significant differences were detected across diet pairs, we conducted post-hoc analysis comparing the intake of nutrients across the sexes within each of the five diet pairs and across all of the diet pairs. In the overall model, the lack of significance of the interaction terms between sex and time and between sex, diet pair and time indicate that the sexes share a common trajectory for the intake of protein and carbohydrates. The lack of significance of the interaction terms between sex and time in the post-hoc models further confirm this conclusion.

|  |  | **Protein** | |  | **Carbohydrate** | |
| --- | --- | --- | --- | --- | --- | --- |
| **Model term** | ***df*** | ***F*** | ***P*** |  | ***F*** | ***P*** |
| Sex (A) | 1,132 | 29.63 | 0.0001 |  | 66.47 | 0.0001 |
| Diet pair (B) | 4,132 | 11.93 | 0.0001 |  | 3.76 | 0.006 |
| Time (C) | 4,129 | 134.10 | 0.0001 |  | 245.58 | 0.0001 |
| A x B | 4,132 | 1.77 | 0.14 |  | 2.09 | 0.09 |
| A x C | 4,129 | 1.83 | 0.13 |  | 2.08 | 0.09 |
| B x C | 16,395 | 4.67 | 0.0001 |  | 4.20 | 0.0001 |
| A x B x C | 16,395 | 1.08 | 0.37 |  | 1.29 | 0.20 |
| **Post-hoc Analysis** |  |  |  |  |  |  |
| *Diet Pair 1* |  |  |  |  |  |  |
| Sex (A) | 1,32 | 6.03 | 0.02 |  | 33.67 | 0.0001 |
| Time (B) | 4,29 | 32.08 | 0.0001 |  | 249.77 | 0.0001 |
| A x B | 4,29 | 1.96 | 0.13 |  | 2.03 | 0.12 |
| *Diet Pair 2* |  |  |  |  |  |  |
| Sex (A) | 1,29 | 0.97 | 0.33 |  | 9.82 | 0.004 |
| Time (B) | 4,26 | 15.06 | 0.0001 |  | 38.10 | 0.0001 |
| A x B | 4,26 | 1.86 | 0.15 |  | 1.46 | 0.24 |
| *Diet Pair 3* |  |  |  |  |  |  |
| Sex (A) | 1,25 | 12.01 | 0.002 |  | 19.37 | 0.0002 |
| Time (B) | 4,22 | 56.22 | 0.0001 |  | 122.80 | 0.0001 |
| A x B | 4,22 | 2.34 | 0.09 |  | 2.05 | 0.12 |
| *Diet Pair 4* |  |  |  |  |  |  |
| Sex (A) | 1,23 | 5.19 | 0.03 |  | 17.47 | 0.0004 |
| Time (B) | 4,20 | 27.29 | 0.0001 |  | 47.94 | 0.0001 |
| A x B | 4,20 | 1.50 | 0.24 |  | 1.93 | 0.14 |
| *Diet Pair 5* |  |  |  |  |  |  |
| Sex (A) | 1,23 | 5.97 | 0.02 |  | 7.43 | 0.01 |
| Time (B) | 4,20 | 14.62 | 0.0001 |  | 20.62 | 0.0001 |
| A x B | 4,20 | 1.75 | 0.18 |  | 1.37 | 0.28 |
| *Across Diet Pairs* |  |  |  |  |  |  |
| Sex (A) | 1,140 | 23.60 | 0.0001 |  | 57.93 | 0.0001 |
| Time (B) | 4,137 | 89.54 | 0.0001 |  | 203.46 | 0.0001 |
| A x B | 4,137 | 1.78 | 0.14 |  | 1.91 | 0.11 |

**Figure Legends**

**Figure S1.** Geometric presentation of the protein and carbohydrate concentration in the 29 holidic diets used in our no-choice experiment (Experiment 1). The individual diets contained within a red circle represent the five diets used in our dietary choice experiment (Experiment 2).

**Figure S2.** Survival curves for female and male flies when consuming diets differing in P:C ratio (2:1, 1:1, 1:2, 1:4, 1:8, 1:16, 0:1 and 0:0), when partitioned by total nutrient concentration (45, 90, 180 and 360 gL^-1^).

**Figure S3.** Mean (±SE) intake of diets in each of the diet pairs for (A) male and (B) female flies. In each diet pair, the protein rich diet is represented by the white bars and the carbohydrate rich diet by the grey bars. The P:C ratio of diets are provided above each bar and the total nutrition of each diet is provided at the base of each bar. A two-factor ANOVA examining the effect of diet pair, sex and their interaction on the difference in intake between the protein and carbohydrate rich diets showed a significant difference across diet pairs (*F*_4,132_ = 33.05, *P* = 0.0001) but not across the sexes (*F*_1,132_ = 0.10, *P* = 0.75) nor was there a significant interaction between diet pair and sex (*F*_4,132_ = 1.14, *P* = 0.34). Paired *t*-tests were used to determine which diet pairs the sexes showed a significant preference for one diet over the other (denoted by an asterisks).

**Figure S4.** Mean (±SE) difference in the intake of protein (white bars) and carbohydrates (grey bars) from the intake of these nutrients if (A) male and (B) female flies fed at random from the two diets in each diet pair. For each fly in our choice experiment (Experiment 2), we measured the total amount of both diets consumed (presented in Figure S3), halved this amount to reflect random feeding on both diets and then calculated the intake of protein and carbohydrate associated with this random feeding. These estimates were then subtracted from the actual intake of P and C on each diet pair for each fly. One sample *t*-tests (testing against a mean of zero) were used to determine if nutrient intake was significantly greater or lower than random feeding (denoted by asterisks).

**Figure S5.** Mean (±SE) absolute intake of protein (black, open symbols) and carbohydrates (grey, closed symbols) of male (A) and female (C) flies when given dietary choice over 5, 3-day feeding intervals, as well as the mean (±SE) number of offspring produced by males (B) and eggs by females (D). Different letters are significant at *P* < 0.05. As flies were mated at 3 days of age, there is no data on reproduction in the first feeding period.

**References**

Piper MDW, Blanc EB, Leitã0-Gonҫalves R, Yang M, He X, Linford NJ, Hoddinott MP, Hopfen C, Soultoukis GA, Niemeyer C, Kerr F, Pletcher SD, Ribeiro C & Partridge L. 2014. A holidic medium for *Drosophila melanogaster*. *Nature Methods* **11**, 100-105.

Sentinella AT, Crean AJ & Bounduriansky R. (2013) Dietary protein mediates a trade-off between larval survival and the development of male secondary sexual traits. *Functional Ecology* **27**, 1134-1144.
